# Radical resection and hyperthermic intraperitoneal chemotherapy (HIPEC) in the treatment of high risk recurrent retroperitoneal sarcoma—A pilot study in a tertiary Asian centre

**DOI:** 10.1371/journal.pone.0300594

**Published:** 2024-04-04

**Authors:** Chin Jin Seo, Joey Wee-Shan Tan, Mohamad Farid, Jolene Si Min Wong, Khee Chee Soo, Claramae Shulyn Chia, Chin-Ann Johnny Ong

**Affiliations:** 1 Department of Sarcoma, Peritoneal and Rare Tumours (SPRinT), Division of Surgery and Surgical Oncology, National Cancer Centre Singapore, Singapore, Singapore; 2 Department of Sarcoma, Peritoneal and Rare Tumours (SPRinT), Division of Surgery and Surgical Oncology, Singapore General Hospital, Singapore, Singapore; 3 Laboratory of Applied Human Genetics, Division of Medical Sciences, National Cancer Centre Singapore, Singapore, Singapore; 4 Division of Medical Oncology, National Cancer Centre Singapore, Singapore, Singapore; 5 SingHealth Duke-NUS Oncology Academic Clinical Program, Duke-NUS Medical School, Singapore, Singapore; 6 SingHealth Duke-NUS Surgery Academic Clinical Program, Duke-NUS Medical School, Singapore, Singapore; 7 Institute of Molecular and Cell Biology, A*STAR Research Entities, Singapore, Singapore; Katholieke Universiteit Leuven UZ Leuven: Katholieke Universiteit Leuven Universitaire Ziekenhuizen Leuven, BELGIUM

## Abstract

**Background:**

Peritoneal sarcomatosis (PS) is a difficult entity to treat with limited options and guarded prognosis. We aimed to determine if the addition of hyperthermic intraperitoneal chemotherapy (HIPEC) could offer superior local recurrence-free survival in patients with retroperitoneal sarcoma at high risk of developing PS as opposed to extended resection alone.

**Methods:**

This is a single arm, phase II intervention study where all patients with recurrent localized retroperitoneal sarcoma considered at high risk of developing PS were considered for enrolment (ClinicalTrials.gov identifier: NCT03792867). Upon enrolment, patients underwent vigorous preoperative testing to ensure fitness for the procedure. During surgery, patients underwent extended resection and HIPEC with doxorubicin. Patients were followed-up every 2 weeks (± 10 days) for the first month and subsequently every three months (± 1 month) up to a year post-surgery, and were assessed for potential chemotherapy toxicity and post-treatment complications. After a year from resection and HIPEC, patients were followed-up either during routine clinic review or contacted via telephone every year (± 1 month) for 3 years.

**Results:**

Six patients were recruited but one patient dropped out due to adverse and unexpected intraoperative events. The remaining patients completed the procedure uneventfully. Post-HIPEC, all patients recurred with a disease-free interval ranging from six to 24 months. Three patients died due to complications from recurrent disease whereas the remaining three patients are alive as of their last visit. The overall survival at time at reporting ranged between 22 to 56 months.

**Conclusion:**

The procedure is feasible with no major morbidity to patients. However, we are unable to recommend for it to be implemented as a routine procedure at this current stage due to lack of improved survival outcomes. Further multi-institutional studies may be conducted to yield better results.

## Introduction

Retroperitoneal sarcomas (RPS) account for 15% of soft tissue sarcomas but only 0.15% of all malignancies [1]. Histologically, the most common RPS subtype is liposarcoma. Liposarcomas can then be further subtyped into three (3) different categories–well differentiated (WDLPS)/dedifferentiated (DDLPS), myxoid/round-cell and pleomorphic LPS [2]. Majority of retroperitoneal liposarcomas will manifest as DDLPS, typically containing high grade segments [3,4].

To date, surgery is the gold standard treatment for localized RPS because of the overall limited effect of pharmacotherapy [5]. Unfortunately, when peritoneal sarcomatosis (PS) develops, there are limited treatment options available and prognosis tends to be dismal [6]. PS is described as the intraperitoneal spread of sarcomas, where pathologically confirmed lesions are detected on the intraperitoneal viscera or peritoneum surface, which are noncontiguous with the primary tumour [7]. This is different from having an RPS component which penetrates the peritoneum and remains contiguous with the main tumour mass. One out of 10 patients with primary RPS will develop PS and patients with PS have an approximate median overall survival of 6–15 months [6–8]. Due to its grim outlook with limited chemotherapy options, surgical methods have to be explored with aims to reduce the occurrence of PS. Even though there are studies that investigate the role of aggressive surgical resection in the hopes for local control of the disease, there is limited information on the use of hyperthermic intraperitoneal chemotherapy (HIPEC) in preventing PS. Most data stem from its use in peritoneal surface malignancies of gynecological, gastrointestinal or mesothelioma origins [9]. Hence, we designed this trial to evaluate the use of radical resection and HIPEC in patients at high risk of developing PS.

In this trial, our primary aim was to determine if prophylactic HIPEC offers superior local recurrence-free survival in RPS patients at high risk of developing PS as opposed to standard treatment, which is extended resection alone. The secondary aim was to compare experimental treatment (prophylactic HIPEC after resection) versus standard resection on overall survival (OS) and disease-free survival (DFS). We also assessed the safety of this experimental treatment with regards to treatment related morbidity and mortality.

## Materials and methods

**Fig 1:**
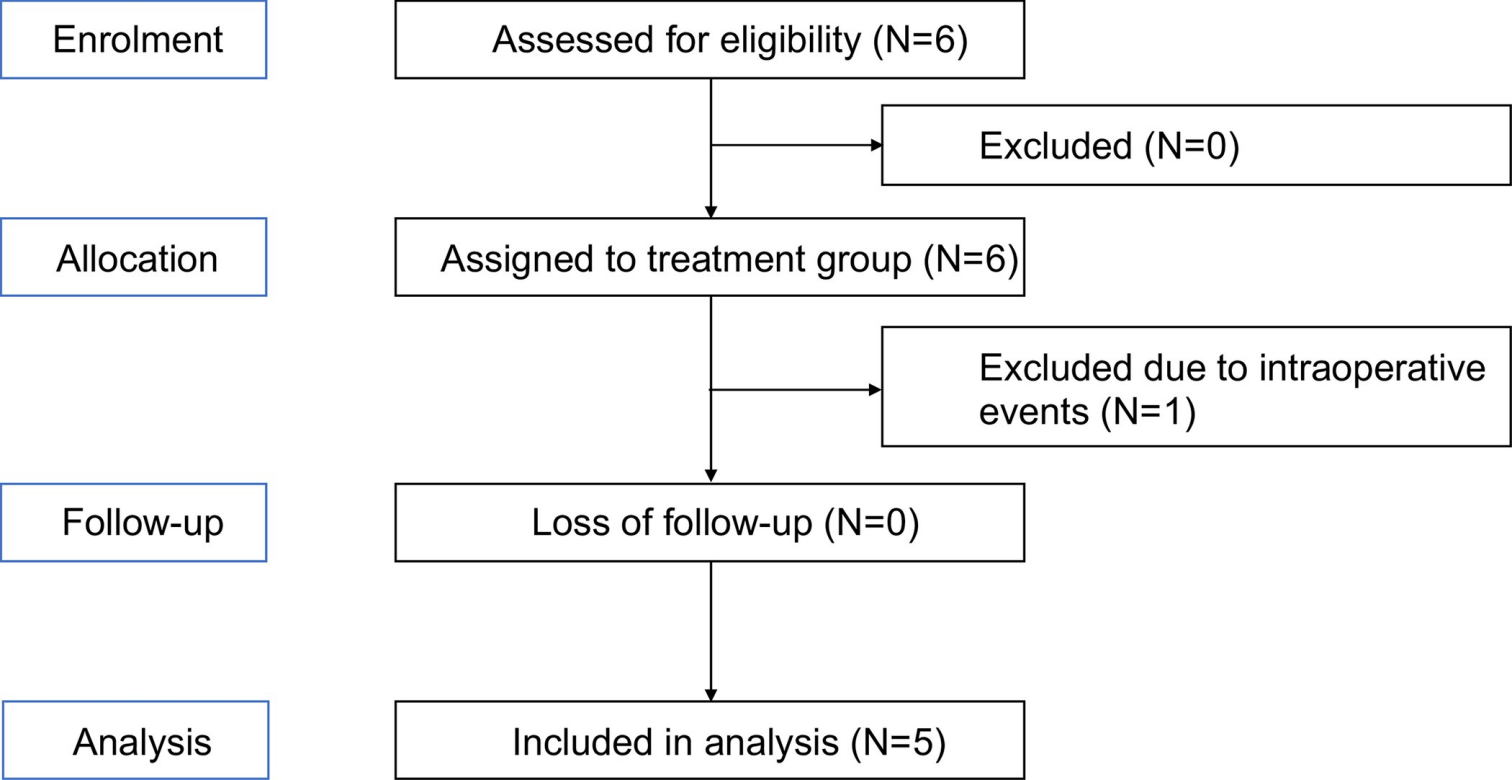
Summary of the flow of participants through each stage (enrolment, intervention, follow-up and analysis) of the study.

This trial is registered under ClinicalTrials.gov (Identifier: NCT03792867) and approved by SingHealth (CIRB No. 2017/2012/B) and Health Services Authorities (HSA). All necessary regulatory requirements were complied with during the course of this study. The flow of participants through each stage of the study is summarized in Fig 1.

### Patient recruitment

This is a single arm, phase II intervention study where patients with sarcoma who were seeking treatment at the National Cancer Centre Singapore (NCCS) and were considered at high risk for developing PS were considered for enrolment. Inclusion criteria for this study were as follows:

- Disease characteristics:
  - Histologically proven soft tissue sarcoma of one of the following high-risk groups:
    - Tumours with either grade 2 or 3 histology
    - Tumour size ≥ 5cm
    - Extracompartmental and deep extension
  - Local recurrence of primary tumour
  - Inadequate surgical excision of previously operated on tumour
  - Proven diagnosis of recurrent retroperitoneal sarcoma confirmed by imaging modality and/or intraoperative biopsy
- Patient characteristics:
  - Age ≥21 years old
  - ECOG performance status of 0–1
  - Normal haematological, hepatic, coagulation, renal and electrolyte profiles
  - Normal left ventricular ejection fraction
  - Not pregnant or nursing

Each case was discussed at our weekly multidisciplinary tumour board meeting to generate a consensus on the suitability of each patient. If the patient was found suitable, they underwent extensive counselling by both the surgical and medical oncology teams regarding possible complications and side effects of the treatment. Patients were recruited from 7 November 2018 to 18 October 2021. Written informed consent was provided by all patients before study enrolment.

### Surgical procedure and HIPEC protocol

Upon enrolment, patients underwent vigorous preoperative testing to ensure fitness for the procedure.

All surgeries were performed by surgeons from the Department of Sarcoma, Peritoneal and Rare Tumours (SPRinT) under general anaesthesia. Selective preoperative prophylactic stenting of the ureters was first carried out if deemed necessary by the primary surgeon. Surgery began with a midline incision extending from xiphoid process to the pubic tubercle. A thorough exploratory laparotomy and exploration of the retroperitoneal area were performed to ensure that there were no other sites of disease. Radical resection was then performed by the surgeon, which typically includes multiple visceral resections directed towards optimal eradication of neoplastic foci from retroperitoneal and involved structures, such as surrounding visceral resection and normal fat prior to instillation of HIPEC. If bowel resection was required, the anastomosis was performed after HIPEC.

Upon complete resection of the tumour, chemotherapeutic agent doxorubicin was instilled into the peritoneal cavity. Peritoneal perfusion was achieved via the formation of a closed-circuit using inflow and outflow catheters. A running suture was used to temporarily close the laparotomy incision in order to establish a watertight seal. Crystalloid solutions were infused until a circuit among the abdominal cavity, pump and heat exchanger was generated. Following the establishment of a good flow, the chemotherapeutic drug, doxorubicin, was instilled into the perfusate and allowed to circulate in the abdominal cavity for 60 minutes. It was diluted into 2–2.5L of peritoneal dialysate fluid at a final concentration of 15 mg/m^2^ and circulated in the abdominal cavity using a Belmont hyperthermia pump, achieving an outflow temperature of 40–42°C. At the end of the HIPEC procedure, the perfusion circuit was drained, the skin reopened and the abdomen lavaged with normal saline. Bowel anastomosis was performed at this juncture if indicated. An average of four drains were left in the abdominal cavity to allow drainage of remnant peritoneal dialysate fluid and the abdominal cavity was closed in the standard fashion.

The drug of choice was doxorubicin, an anti-tumour antibiotic drug that exerts cytotoxicity via topoisomerase II inhibition, DNA intercalation and formation of reactive oxygen free radicals [10]. Systemic doxorubicin has demonstrated efficacy in a wide range of sarcoma histologic subtypes and is a widely accepted standard first line agent in advanced disease [11]. Moreover, doxorubicin has multiple properties that makes it safe to use as a HIPEC agent, namely, it is stable and acts synergically with hyperthermia, has a high area under the curve (AUC) ratio of intraperitoneal to plasma compartment, and confers to the single pass hepatic metabolism that decreases risk of systemic toxicity [12]. The main side effect of concern is that of peritoneal inflammation, sclerosis and intestinal obstruction [12]. Dose escalation studies have been performed by Sugarbaker and colleagues which demonstrated that a dose of 15mg/m^2^ is safe with regards to subsequent gastrointestinal function [13].

### Follow-up care and surveillance

Following surgery, patients were followed-up every 2 weeks (± 10 days) for the first month and subsequently every three months (± 1 month) up to a year post-surgery. During the follow-up session, patients were assessed for potential chemotherapy toxicity and post-treatment complications. Adverse events were captured and addressed at the discretion of the examining doctors. Assessments performed during the follow-up sessions included, but are not limited to, routine blood count, renal panel and liver function tests. Records of adjuvant treatments or treatments upon recurrence of disease were also collected.

After a year from resection and HIPEC, patients were followed-up either during routine clinic review or contacted via telephone every year (± 1 month) for 3 years.

### Sample size calculation

Study sample size population was based on the hypothesis that radical resection and HIPEC could reduce the recurrence rate of PS from 80% to 60%. Using a power of 0.9, alpha of 0.05, one sided test, an estimated standard deviation difference of about 2.25 and the disease-free interval (DFI) difference of 1.5 months between those receiving HIPEC and without HIPEC, the sample size required was 21 patients.

## Results

### Demographic and baseline clinical characteristics of recruited patients

Six patients were recruited from 7 November 2018 to 18 October 2021. All patients were diagnosed with recurrent localized RPS on follow-up. Their demographics are as detailed in Table 1.

**Table 1:** Baseline clinical characteristics of recruited patients.

Characteristics	No (N = 6)
**Age**	34, 37, 49, 56, 59, 76
**Gender**	Female 4: Male 2
**Race**	Chinese 5: Malay 1
**ASA** • **I** • **II** • **III** • **IV**	0 4 2 0
**Comorbidities** • **Hypertension** • **Hyperlipidaemia** • **Chronic kidney disease** • **Diabetes** • **Ischaemic heart disease** • **Others**	1 2 2 2 0 2 –Graves disease(1), Papillary thyroid cancer (1)

Abbreviations: ASA, American Society of Anesthesiologists

None of the patients received cytotoxics prior to trial enrolment. Only one patient had undergone two surgeries for recurrent retroperitoneal sarcoma prior to trial enrolment and had a course of ‘neoadjuvant’ radiotherapy to the surgical bed prior to HIPEC surgery. Prior to enrolment, every patient’s clinical history was discussed at the multidisciplinary tumour board, consisting of medical oncologists, radiation oncologists, surgical oncologists, and radiologist.

In this study, the index retroperitoneal sarcoma surgery were performed at various institutions, by surgeons of various subspecialities such as obstetrics and gynaecology. Once diagnosis of sarcoma is confirmed, they will then be referred over to our tertiary subspecialty unit. We were able to obtain all surgical notes and histological reports for this study. The histology of all patients was that of liposarcoma. Two patients had WDLPS at initial diagnosis, but on recurrence had been upstaged to DDLPS. The remaining three patients had DDLPS at outset, with a baseline FNCLCC Grade of 2 to 3.

At initial surgery, patients generally had multivisceral resection and this may include one or any of the following—right or left hemicolectomy, nephrectomy, resection of part of the diaphragm, and distal pancreatectomy and splenectomy. When multivisceral resection was performed, it was done en bloc with the tumour itself to ensure there was no breech of the tumour capsule. The size of the initial resected tumour varied from 9 to 39cm and majority had R1 resection according to the final histopathology report. Table 2 details the disease characteristics of patients at index and post recurrence surgery with HIPEC.

**Table 2:** Disease characteristics of patients at index and post recurrence surgery with HIPEC.

Disease Characteristics	Index surgery	HIPEC Surgery
**Number of patients**	N = 6	N = 5
**Histology** • **Well differentiated** • **Mixed well and dedifferentiated** • **Dedifferentiated**	2 1 3	0 1 4
Initial FNCLCC Grade • **1** • **2** • **3** • **Not available**	1 3 2 0	0 2 0 3
**Tumour size (cm)**	9,7,20,20,33,39	4,5.1,5.8,9,17
**Viscera resected** • **Colon** • **Kidney** • **Salphingoopherectomy** • **Diaphragm** • **Distal pancreas** • **Spleen**	3 4 1 1 1 1	-
**Resection margin status** • **R0** • **R1** • **R2**	1 5 0	1 4 0
**Recurrence** • **Locoregional** • **Distant**	6 0	2 3

### Study intervention: Intraoperative and postoperative details

Among the six recruited patients, only five underwent HIPEC successfully. Operative times ranged from 220–490 minutes (median 330 minutes) with a median blood loss of 500mls (range 300-1000mls). Post operatively, only one patient required intensive care unit monitoring for one day to ensure stability and was subsequently transferred to the high dependency unit for monitoring the following day. The remaining four patients were monitored in the high dependency unit until deemed safe by the primary surgeon to step down to the general ward for continued care. Surgical drains were removed when volume and content of drainage was appropriate. Diet was escalated accordingly and no patient had prolonged ileus requiring total parenteral nutrition support. Hospitalisation stay ranged from 5–12 days (median 6 days).

### Adverse events

We had one patient who did not receive HIPEC and was dropped out of the study. The patient had undergone initial retroperitoneal liposarcoma resection in April 2019. The initial histology was that of a dedifferentiated liposarcoma measuring 33cm, with R1 resection margins. Post surgery she was discussed at the tumour board and decision was made for adjuvant chemotherapy alone in view of the possible morbidity associated with wide-field radiotherapy. She completed 6 cycles of doxorubicin and ifosfamide. Unfortunately, local recurrence was noted in September 2020 during her follow up scan, at the left iliac fossa region. Her case was discussed, she was enrolled and we proceeded with resection of the recurrent tumour. Intraoperatively, the tumour was extremely adherent to the left internal iliac artery and a segment had to be resected en bloc with the tumour. During dissection there was inadvertent injury to the left internal and external iliac veins resulting in an estimated blood loss of 5.1L causing haemodynamic instability despite massive transfusion. Decision was then made not to proceed with HIPEC as the bleeding was unexpected and safety concerns for the patient preceded the study protocol (S1 File).

Following HIPEC, another patient experienced an adverse event (Clavien Dindo II). The patient was readmitted 19 days post-operation for fever and abdominal pain. A computed tomography of the abdomen showed pneumoperitoneum, suggestive of an anastomotic leak. As the patient was clinically stable and not peritonitic, the decision was made for conservative treatment with antibiotics and total parenteral nutrition. Follow-up scans showed resolution with no further contrast leak and resolution of the pneumoperitoneum. The patient recovered well and was discharged with no further complications. No further patients developed severe adverse event (Clavien Dindo III/IV).

### Survival outcomes

The 90-day post-operative mortality rate for this study was 0%.

Final histology confirmed DDLPS for all resected recurrences. Only one patient had R0 resection, the rest had R1 resection margins confirmed on histology. Despite the R0 resection during the radical surgery and HIPEC, this patient only had an 11-month disease free interval and has since recurred.

**Fig 2:**
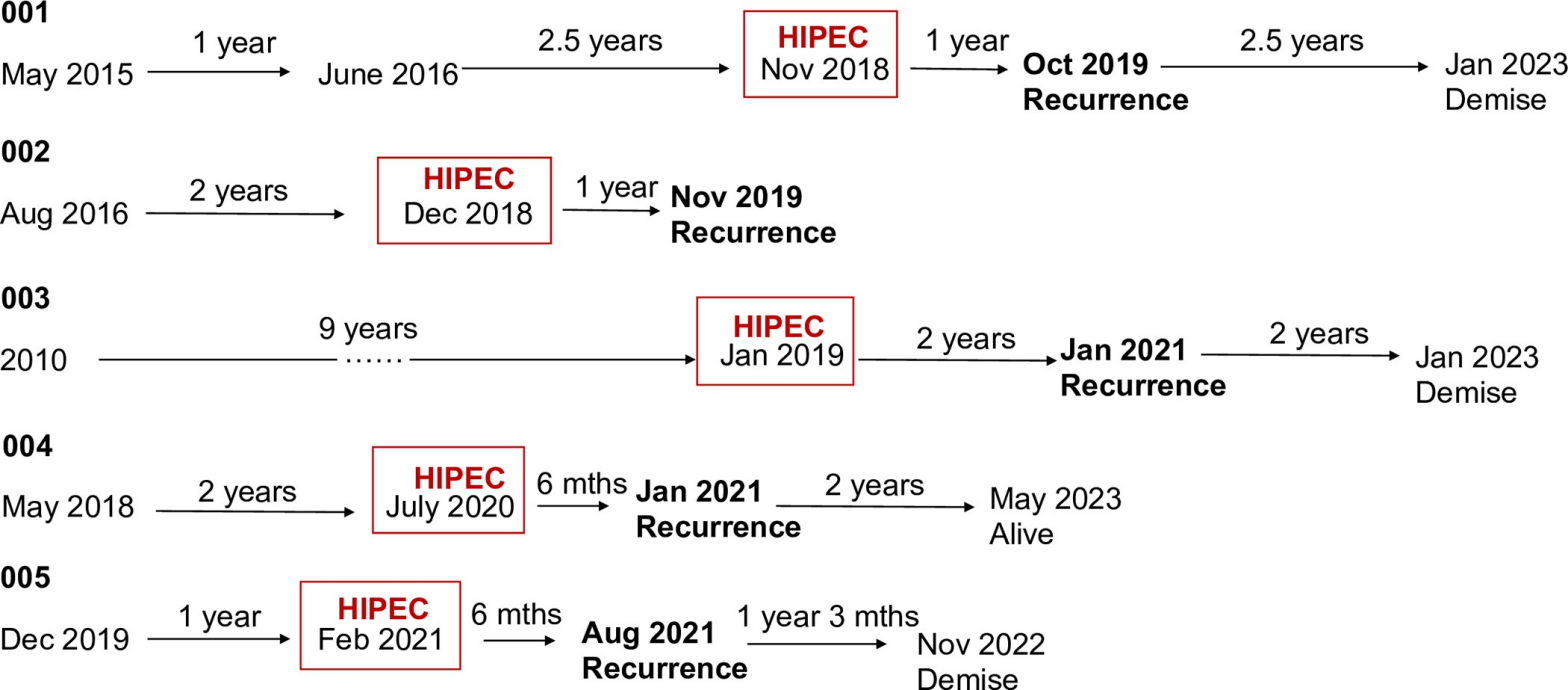
Pictorial representation of the significant events of the recruited patients.

The calculated disease-free interval (DFI) post HIPEC ranges from 6 to 24 months (Fig 2). Three patients have died due to complications from recurrent disease whereas two patients are alive as of last follow-up. The calculated overall survival (OS) ranged between 22 and 56 months.

In view of the rarity of the disease, we compared the survival outcome of these patients to an unmatched cohort with recurrent retroperitoneal liposarcomas who was treated at our institution between 2002 and 2021, whose data was retrieved as part of an internal department audit. DFI to first recurrence was a median of 20 months (IQR 9–31). DFI between each recurrence got progressively shorter, where it was a median of 13 months (IQR 4–20) for second recurrence and a median of 7 months (IQR 1–11) for third recurrence. With each recurrence, we also note that a significantly higher proportion developed peritoneal sarcomatosis and metastatic disease, with higher grade dedifferentiated tumours.

## Discussion

Soft tissue sarcomas are a rare form of mesenchymal tumours making up only 1% of all malignancies and typically metastasize haematogenously to lungs, liver or directly to peritoneal surfaces and adjacent organs [14,15]. The two most common subtype of soft tissue sarcoma would be liposarcoma followed by leiomyosarcomas. Liposarcomas have been recognized to exhibit a high recurrence potential in primary disease. WDLPS lack metastatic potential but almost always recur locally whereas DDLPS recur both locally and distally [16]. Even in the presence of just WDLPS, recurrence of the tumour in inadequately excised primary shows a potential to dedifferentiate and metastasize [17,18]. Due to its anatomic location in the retroperitoneum where it is in close proximity to major critical structures, there is a potential risk of positive microscopic margins during primary resection. The rates of positive microscopic margins also increase progressively with increasing primary tumour size, where tumours >20cm had a 43% risk of recurrence as compared to those <5% where the risk was only 13% [17]. This in turn correlates with higher rate of distant recurrence and consequently lower recurrence-free survival as shown in a univariate and multivariate analysis done by Stojadinovic et al. [17].

Leiomyosarcomas on the other hand are considered distally aggressive tumours with a propensity for hematogenous dissemination. Though survival with conventional treatments is poor, some evidence may suggest intra-abdominal leiomyosarcoma exhibits a better response to CRS-HIPEC, especially in the setting of uterine leiomyosarcomas [19,20]. However, the reason behind the difference in response for each subtype towards CRS and HIPEC is poorly understood.

There are currently no clear guidelines for the management of recurrent RPS with high risk of PS. To date, limited data is available owing to the poor prognosis of these patients. We do note that studies have been done investigating the role of radical surgery and HIPEC in established peritoneal sarcomatosis such as those performed by Randle et al. and Baumgartner et al. [21,22]. However, as expected, sample size was small and mixed histologies were included, making it difficult to reach any definite conclusion on the role of HIPEC. A meta-analysis of CRS HIPEC in patients with established PS also suggested that CRS HIPEC may augment the outcomes of highly selected patients [23]. Then again, the study included mainly retrospective single armed publications as well as sarcomas of heterogenous histology, such as leiomyosarcoma, liposarcoma and gastrointestinal stromal tumour (GIST). Hence, despite the available evidence, there is no established utility of CRS HIPEC in PS, let alone in the prevention of sarcomatosis in high risk recurrent RPS.

In view of the above, we decided to perform an investigation on the role of HIPEC in high-risk patients, extrapolating the indication from those of peritoneal surface malignancies, for example, the OVHIPEC trial where implementation of interval CRS and HIPEC after neoadjuvant chemotherapy achieved improved recurrence-free survival and overall survival [24]. Unfortunately, following recruitment of six patients, the study was prematurely terminated in view of poor accrual and a lack of signal during analysis.

The gold standard treatment for primary localized retroperitoneal sarcoma is extended resection so as to augment local control [25]. To determine the importance of resection in sarcoma treatment, Guo et al. [26] performed a meta-analysis of survival outcomes of patients with primary and recurrent retroperitoneal sarcoma following resection. Their study population included all patients who underwent surgery. Overall outcomes showed that as long as the patient underwent surgery, regardless of the extent of resection and margins, survival outcomes were better than those who were treated conservatively. In a subgroup analysis of primary RPS, R0 resection was found to be better than R1 resection, and subsequently R1 resection was superior to R2 resection.

In our current study, we demonstrated that HIPEC was both safe and feasible in patients at high risk of developing PS. However, many patients with recurrent retroperitoneal sarcomas may have developed metastasis to distant organs, such as the lungs, rendering them ineligible for surgery. This poses a challenge in organizing and recruiting patients for randomized controlled trials to determine the true efficacy of HIPEC in these patients. A multicenter trial would have to be conducted, with standardization of patient selection and HIPEC protocol, in order to recruit more patients to get a better understanding of the disease.

Despite having undergone complete cytoreduction and HIPEC, all our patients have developed recurrent sarcoma, with DFI ranging between 6 to 24 months. Moreover, three of five patients have demised. There are many studies describing the recurrence risk after primary resection. For example, a study from Macneil et al. that looked at the relapse rates following primary extended resection of RPS reported a median recurrence time of about 41 months [27]. Another study done at John Hopkins showed that median relapse free interval (RFI) for any recurrence in patients that underwent a R0 resection was 35.9 months as opposed to 18.2 months for those who had a R1 resection [28]. However, very few studies describe the risk of recurrence after a redo resection for retroperitoneal sarcomas. A small study from Korea reported that with every repeat surgery for recurrent retroperitoneal sarcoma, the relapse free interval shortens significantly, from 25 months RFI after second surgery to 15 months RFI after a third surgery, and down to 5 months RFI after a fifth operation [29]. Similarly, this observed trend is reflected in our study (Fig 2).

With regard to the patient who experienced an adverse event, we are unable to prove that the complication happened due to the addition of HIPEC to the surgery as we know that occult anastomotic leak could happen after a major surgery. In general, all patients did not suffer severe post-operative morbidity and 90-day post-operative mortality for our patients stand at 0%. Hence, this is a relatively safe procedure based on our small sample size.

Another point to note is that even though the study coincided with the COVID 19 pandemic, our institution was minimally affected. Patients were still able to seek prompt medical attention and there was no restriction in management, especially surgical intervention of patients with proven malignancy as this was a time sensitive condition. Hence the impact on accrual of patients and their subsequent follow up was minimal. None of the patients recruited passed on due to COVID.

Taken together, the utility of HIPEC in addition to extended resection for patients at high risk of developing PS remains debatable. A deeper understanding of tumour biology, such as the use of genomic or transcriptomic analyses to correlate patient susceptibility for targeted therapy, may provide better outcomes. The fluid paracrine tumour microenvironment could also offer clues to the identification of novel therapies for these patients [30]. Based on our study, the utility of HIPEC in this setting to prevent peritoneal sarcomatosis cannot be concluded. Despite this, we have attempted to analyse the outcomes of this small group of patients to assess the feasibility HIPEC surgery. As we did not achieve an outcome supporting our hypothesis, we decided to abort the study early. Nevertheless, we do believe that by publishing our results, we will be able to shed more light on this issue and help the direction of future studies in this area.

## Conclusion

This study did not show an improved disease-free survival or prevent the development of peritoneal sarcomatosis in patients with high-risk recurrent retroperitoneal sarcoma. However, it does show that the surgical procedure itself is feasible with acceptable post operative morbidity and outcomes. Future studies may require multi-institutional involvement to improve accrual rates.

## Supporting information

S1 Checklist The TIDieR (Template for Intervention Description and Replication) checklist*. (DOCX)

S2 Checklist TREND statement checklist. (PDF)

S1 Data (XLSX)

S1 File Study protocol. (PDF)
