# Supplementary material for: Radical resection and hyperthermic intraperitoneal chemotherapy (HIPEC) in the treatment of high risk recurrent retroperitoneal sarcoma—A pilot study in a tertiary Asian centre
Source: PLoS One. 2024 Apr 4;19(4):e0300594. doi: 10.1371/journal.pone.0300594 (PMC10994346; doi:10.1371/journal.pone.0300594)
Supplement: S1 File — (PDF) [file pone.0300594.s004.pdf]

CLINICAL TRIAL PROTOCOL

PROTOCOL TITLE:
Radical resection and Hyperthermic Intraperitoneal Chemotherapy (HIPEC) in the treatment of high risk recurrent retroperitoneal sarcoma

PROTOCOL NUMBER: 2017/2012/B

PROTOCOL VERSION: 6.0
PROTOCOL DATE: 21 Feb 2020

PRINCIPAL INVESTIGATOR:
Dr Johnny Ong Chin-Ann, Consultant, National Cancer Centre Singapore

CO-INVESTIGATORS:
Dr Melissa Teo Ching Ching, Visiting Senior Consultant, National Cancer Centre Singapore
Dr Mohammad Farid Bin Harunal Rashid, Consultant, National Cancer Centre Singapore
Dr Richard Quek, Senior Consultant, National Cancer Centre Singapore
Prof Soo Khee Chee, Visiting Consultant, National Cancer Centre Singapore
Dr Claramae Chia, Consultant, National Cancer Centre Singapore
Dr Grace Tan, Consultant, National Cancer Centre Singapore
Dr Jolene Wong Si Min, Associate Consultant, Singapore General Hospital

STUDY TEAM MEMBERS:
Ms Qiu Xuan, Research Officer, National Cancer Centre Singapore
Ms Joey Tan Wee Shan, Research Associate, National Cancer Centre Singapore
Dr Lek Sze Min, Medical Officer, MOHH
Dr Selvarajan Sathiyamoorthy,Senior Consultant, Singapore General Hospital
Dr Tay Kwang Yong Timothy, Consultant, Singapore General Hospital
Ms Chong Hui Shan, Clinical Research Coordinator, National Cancer Centre Singapore
Ms Lim Tze Wei, Clinical Research Coordinator, National Cancer Centre Singapore
Ms Stella Chan Li Li, Clinical Research Coordinator, National Cancer Centre Singapore
Ms Lee Xiao Wen, Clinical Research Coordinator, National Cancer Centre Singapore
Ms Cristal Bacala Laurente, ACRC, National Cancer Centre Singapore
Ms Goh Jie Xuan, Clinical Research Coordinator, National Cancer Centre Singapore
Ms Kor Hui Min, Clinical Research Coordinator, National Cancer Centre Singapore
Ms Sylvia Ong Xue Ting, Clinical Research Coordinator, National Cancer Centre Singapore
Ms Gillian Ng Wen Xuan, Research Technologist, National Cancer Centre Singapore
Ms Lisa Mak, Associate Executive, National Cancer Centre Singapore

Mr Lew Kuang Yuan, Pharmacist, National Cancer Centre Singapore
Ms Goh Ai Hui Ivy, Pharmacist, National Cancer Centre Singapore
Ms Ng Hui Cheng, Pharmacist, National Cancer Centre Singapore
Ms Sia Hwee Leng, Pharmacist, National Cancer Centre Singapore
Ms Padilla Krister, Pharmacist, National Cancer Centre Singapore

Table of Contents

1 BACKGROUND AND RATIONALE 5

1.1 GENERAL INTRODUCTION 5
1.2 RATIONALE AND JUSTIFICATION FOR THE STUDY 6
1.2.1 RATIONALE FOR THE STUDY PURPOSE 6
1.2.2 RATIONALE FOR DOSES SELECTED 6
1.2.3 RATIONALE FOR STUDY POPULATION 7
1.2.4 RATIONALE FOR STUDY DESIGN 7

2 HYPOTHESIS AND OBJECTIVES 7

2.1 HYPOTHESIS 7
2.2 PRIMARY OBJECTIVES 7
2.3 SECONDARY OBJECTIVES 7
2.4 POTENTIAL RISKS AND BENEFITS: 7
2.4.1 POTENTIAL RISKS 7
2.4.2 POTENTIAL BENEFITS 8

3 STUDY POPULATION 8

3.1 TARGET POPULATION 8
3.2 CRITERIA FOR RECRUITMENT AND RECRUITMENT PROCESS 8
3.3 INCLUSION CRITERIA 8
3.4 EXCLUSION CRITERIA 9

4 STUDY DESIGN 9

4.1 SCREENING VISITS AND PROCEDURE 9
4.2 RADICAL RESECTION 9
4.3 HYPERTHERMIC INTRAPERITONEAL CHEMOTHERAPY (HIPEC) 10
4.4 FOLLOW-UP VISITS 10
4.5 SURVIVAL FOLLOW-UP 11
4.6 DISCONTINUATION/WITHDRAWAL 11
4.6.1 DISCONTINUATION CRITERIA 11
4.6.2 DISCONTINUATION VISIT AND PROCEDURES 11

5 CHEMOTHERAPY DRUG 11

5.1 RATIONALE FOR SELECTION OF DOSE 11
5.2 STUDY DRUG ADMINISTRATION 12
5.3 STORAGE AND DRUG ACCOUNTABILITY 12

6 SAFETY MEASUREMENTS 12

6.1 DEFINITIONS 12
6.2 COLLECTING, RECORDING AND REPORTING OF ADVERSE EVENTS AND SERIOUS ADVERSE EVENTS TO CIRB 13
6.3 COLLECTING, RECORDING AND REPORTING OF SERIOUS ADVERSE EVENTS (SAES) TO THE HEALTH SCIENCE AUTHORITY (HSA) 13
6.4 SAFETY MONITORING PLAN 13

6.5 COMPLAINT HANDLING 13

7 DATA ANALYSIS 14

7.1 DATA QUALITY ASSURANCE 14
7.2 DATA ENTRY AND STORAGE 14

8 SAMPLE SIZE AND STATISTICAL METHODS 14

8.1 DETERMINATION OF SAMPLE SIZE 14
8.2 STATISTICAL AND ANALYTICAL PLANS 15

9 DIRECT ACCESS TO SOURCE DATA/DOCUMENTS 15

10 QUALITY CONTROL AND QUALITY ASSURANCE 15

11 ETHICAL CONSIDERATIONS 15

11.1 INFORMED CONSENT 16
11.2 CONFIDENTIALITY OF DATA AND PATIENT RECORDS 16

12 PUBLICATIONS 16

13 RETENTION OF TRIAL DOCUMENTS 16

14 FUNDING AND INSURANCE 16

PROTOCOL SIGNATURE PAGE

Protocol Title: Radical resection and Hyperthermic Intraperitoneal Chemotherapy (HIPEC) in the treatment of high risk recurrent retroperitoneal sarcoma

Protocol Number: 2017/2012/B

Protocol Version/ Date: 6.0 / 21 Feb 2020

Declaration of Investigator

I confirm that I have read the above-mentioned protocol and its attachments. I agree to conduct the described trial in compliance with all stipulations of the protocol, regulations and ICH E6 Guideline for Good Clinical Practice (GCP).

Principal Investigator Name: Dr Johnny Ong Chin Ann

Principal Investigator Signature: ______________________________________

Date: ____________________

1 BACKGROUND AND RATIONALE

1.1 General Introduction

Retroperitoneal sarcomas account for 15% of soft tissue sarcomas and approximately 50% of patients will develop local or locoregional recurrence regardless of therapy [1-3]. Current management strategy is surgical resection [4-7]. However, the extent of surgical resection has been widely debated with no conclusive guidelines although complete resection with clear margins has been shown to have improved survival [1, 8]. Furthermore, retroperitoneal sarcomas have poor response to chemo-radiotherapy with limited evidence for loco-regional control but no improvement in overall survival [9, 10]. Recent studies have shown promise where aggressive surgical resection with localised hyperthermia and systemic chemotherapy provides for better local control and lower local recurrence rates in locally recurrent retroperitoneal sarcomas [11, 12]. Furthermore, there has been increased interest in performing cytoreductive surgery with hyperthermic intraperitoneal chemotherapy for the treatment of peritoneal sarcomatosis [13-17]. Thus, this study investigates the use of radical resection and hyperthermic intraperitoneal chemotherapy in high risk recurrent retroperitoneal sarcoma. In addition, we seek to evaluate its efficacy in improving recurrence-free and overall survivals as well as unforeseen toxicities from the use of hyperthermic intraperitoneal chemotherapy and the proposed chemotherapeutic drugs.

1.2 Rationale and Justification for the Study

Retroperitoneal sarcomas have a high risk of local or regional recurrence with a recurrence rate of 40-60% after complete resection. Current treatment, which involves surgical resection, has not significantly improved recurrence rates or overall survival. In studies specifically addressing resection of locally recurrent retroperitoneal sarcoma, complete resection was significantly associated with increased survival when en-bloc resection of adjacent organs and tissue is performed versus only complete gross tumour resection.

Issels and colleagues recently conducted a phase 3 multicentre trial on patients with localised high-risk soft tissue sarcomas (STS), including locally recurrent retroperitoneal sarcoma, who were randomized into receiving either neo-adjuvant chemotherapy alone or combined with regional hyperthermia in addition to local therapy [11, 12]. Aggressive surgical resection with localised hyperthermia and systemic chemotherapy provided an effective treatment strategy for patients with high-risk STS, improving local tumour control and lowering local recurrence rates. Furthermore, there has been increased interest in performing cytoreductive surgery with HIPEC for the treatment of peritoneal sarcomatosis, although the role of HIPEC in improving patient survival is uncertain [13-19]. We have recently published a case report on a patient with peritoneal sarcomatosis managed with this combined approach [20]. Extrapolating from the evidence cited, we propose the use of complete resection coupled with HIPEC in the context of complete removal of disease to decrease loco-regional recurrence in retroperitoneal sarcoma.

The HIPEC drug to be used in this clinical trial is doxorubicin only. Doxorubicin is an anti-tumour antibiotic that exerts cytotoxicity via topoisomerase II inhibition, DNA intercalation and formation of reactive oxygen radicals [21]. Systemic doxorubicin has demonstrated efficacy in a wide range of sarcoma histologic subtypes and is a widely accepted standard first line agent in advanced disease [22]. Doxorubicin has several attributes that render it suitable for intraperitoneal use, including stability and synergism with hyperthermia, high AUC ratio of intraperitoneal to plasma compartment, and single pass hepatic metabolism that decreases risk of systemic toxicity [23].

Therefore, this project aims to determine the effectiveness of radical surgical resection in combination with HIPEC in treating patients with recurrent retroperitoneal sarcoma by improving local control and progression free survival.

1.2.1 Rationale for the Study Purpose

Recurrent retroperitoneal sarcoma often represents poor prognosis and does not respond well to systemic chemotherapy [9, 10]. In treatment of sarcoma, radical resection with clear margins is essential to prevent recurrence. Radical surgery ensuring clear surgical margins and taking cuffs of normal tissue/fat/muscle is hence the standard of care surgery [4-7]. Recent trials involving hyperthermia and/or radiation therapy have shown to improve local control [11, 12]. It is not known whether radical surgical resection in combination with hyperthermic intraperitoneal chemotherapy (HIPEC) would be effective in treating patients with recurrent retroperitoneal sarcoma by improving local control and progression free survival.

1.2.2 Rationale for Doses Selected

The HIPEC dose of doxorubicin to be used in this study is 15mg / m2. Systemic doxorubicin has demonstrated activity and efficacy in a broad spectrum of soft tissue sarcomas [22]. It has also been evaluated in the intraperitoneal setting, in which setting the chief toxicity of concern is that

of peritoneal inflammation and sclerosis and intestinal obstruction. Dose escalation studies performed by Sugarbaker and colleagues revealed a dose of 15mg/m2 to be safe with regards to subsequent gastrointestinal function [24].

The duration of doxorubicin to be used in this study is 60 minutes. HIPEC is usually performed for 60 – 90 minutes after resection is completed [23]. In our institution, HIPEC is administered for 60 minutes in all cases [25].

1.2.3 Rationale for Study Population

The National Cancer Centre Singapore is a tertiary centre that sees the majority of the patients with cancer within the country, many of whom have recurrent sarcomas. These patients typically have a poor prognosis and have a high risk of further recurrences.

1.2.4 Rationale for Study Design

This single arm interventional study is designed to determine the overall effectiveness and efficacy of this treatment modality as compared with current management for high risk recurrent retroperitoneal sarcoma and to evaluate any unforeseen toxicities of this treatment.

2 HYPOTHESIS AND OBJECTIVES

2.1 Hypothesis

We hypothesize that radical resection and hyperthermic intraperitoneal chemotherapy will improve loco-regional control and improve recurrence- free survival and potentially, overall survival. Patients with recurrent sarcomas can benefit from undergoing radical resection and HIPEC whereby better loco-regional control could improve recurrence- free survival and potentially overall survival.

2.2 Primary Objectives

The primary objectives of this study are to determine the feasibility of conducting radical resection and HIPEC in patients with retroperitoneal sarcoma and to evaluate the chemotherapy toxicity and unforeseen acute and late complications associated with radical resection and HIPEC in the treatment of patients with high risk retroperitoneal sarcoma.

2.3 Secondary Objectives

The secondary objectives of this study are to determine if the introduction of radical surgery and HIPEC would improve the local recurrence-free and overall survivals of patients with high risk retroperitoneal sarcoma as compared to current treatment strategies.

2.4 Potential Risks and Benefits

2.4.1 Potential Risks

Potential risks to patients would include unforeseen complications of surgical resection and hyperthermic intraperitoneal chemotherapy. These specific techniques have been well studied in its use for treatment in other cancers and its complications can be reasonably anticipated and managed in the clinical scenario [13-17, 26-28].

Expected surgical risks will include bleeding, infection, anastomotic leaks as well as surgical complications requiring further surgical interventions. Potential risks of hyperthermic intraperitoneal chemotherapy with the chemotherapy agent, doxorubicin, include risk of developing inflammation of the abdominal cavity that can result in pain, intestinal obstruction and impaired nutrition. There is a risk of chemotherapy entry into the general circulation during or immediately after surgery in which systemic side effects associated with doxorubicin can develop. Short term toxicities include nausea and vomiting, lowered blood cell counts, fatigue, infections, risk of bleeding, constipation, diarrhoea, sores of mouth and throat, hair loss and damage to nerves. Long term systemic toxicities include fertility loss, damage to heart function and secondary cancers of the blood. In the event of severe complications from chemotherapy, this could be life-threatening and cause death.

2.4.2 Potential Benefits

As this study is experimental, there is no potential benefit guaranteed to the patient.

3 STUDY POPULATION

3.1 Target population

A total of 50 patients will be recruited from the National Cancer Centre Singapore. Eligible patients will undergo surgery and HIPEC at the Operating Theatre at the satellite site - Singapore General Hospital. There is no subject restriction based on race or gender of the patient. At screening, potential patients will be assigned a screening number. Following confirmation of eligibility, patients will be assigned to a 4-digit patient number. Patients who are screen failure or have dropped out of the study will be replaced.

3.2 Criteria for Recruitment and Recruitment Process

Patients recruited will have to be identified and diagnosed with high risk recurrent retroperitoneal sarcoma.

3.3 Inclusion Criteria

The patient must meet all of the inclusion criteria to participate in this study.

a. DISEASE CHARACTERISTICS:
- Histologically proven soft tissue sarcoma of one of following high-risk groups:
  i. Tumours with grade 2 or 3 histology
  ii. Size more than or equal to 5cm
  iii. Extracompartmental and deep extension
- Local recurrence of primary tumor
- Inadequate surgical excision of previously operated on tumour
- Proven diagnosis of recurrent retroperitoneal sarcoma confirmed by imaging modality and/or intraoperative biopsy

b. PATIENT CHARACTERISTICS:
- Age: ≥21 years old
- Performance status: ECOG 0-1

- Normal haematological, hepatic, coagulation, renal and electrolyte profiles
- Normal left ventricular ejection fraction
- Not pregnant or nursing

3.4 Exclusion Criteria

a. Patient is medically unfit for surgery due to concurrent medical comorbidities.
b. Any medical or psychiatric condition(s) which would preclude informed consent.
c. Patient is pregnant or nursing.

4 STUDY DESIGN

Screening → Radical resection → HIPEC (Doxorubicin) → Follow-up for toxicity → Survival follow-up

4.1 Screening Visits and Procedure

Screening begins after the patient signs the informed consent form. The screening ends with confirmation of full eligibility to participate in this clinical trial or confirmation that the patient is a screen failure. All screening visits and procedures are done according to routine screening for all subjects presenting for treatment at the institution. Prior to surgery, patients would undergo a 2D-Echocardiography to assess his/her cardiac status. All investigations performed are routine in preparation for operation, including but not limited to haematological, hepatic, coagulation, renal, electrolyte, tumour markers profiles.

For female patients of child-bearing potential (i.e., those who are not post-menopausal for at least 1 year or surgically sterile by bilateral salpingectomy, bilateral oophorectomy or hysterectomy), a urine pregnancy test will be performed before the resection. Pregnancy should be avoided during the trial with the use of reliable contraception.

4.2 Radical Resection

The patient will receive surgery i.e. radical resection and hyperthermic intraperitoneal chemotherapy as part of the planned treatment. All cases are discussed at a multidisciplinary meeting involving surgical oncologists, medical oncologists, radiation oncologists and relevant involved parties. Options of surgical extent and subsequent therapy with neo-adjuvant or adjuvant chemotherapy or radiotherapy will be discussed and determined. All operations are carried out by the same surgeons from the Division of Surgical Oncology.

Under general anaesthesia, justified prophylactic (not routine) ureteral stents will be inserted by the urology team at the beginning of the operation before carrying out radical resection plus HIPEC procedure. If a bowel resection is required, anastomosis will be performed after HIPEC. A midline incision extending from xiphoid process to pubic tubercle will be performed to completely explore the retroperitoneal cavity for the recurrent retroperitoneal sarcoma. The extent of disease is determined and radical resection is performed. This includes multiple visceral

resections directed towards optimal eradication of neoplastic foci from the retroperitoneal and involved structures including but not limited to surrounding visceral resection and normal fat.

4.3 Hyperthermic Intraperitoneal Chemotherapy (HIPEC)

Closed abdomen HIPEC technique will be performed at the end of radical resection. The open cavity will be lavaged with normal saline prior to HIPEC. Peritoneal perfusion is achieved by a closed circuit with inflow and outflow catheters placed through the skin. The laparotomy incision will be closed with a running suture at the skin level to create a watertight seal. Crystalloid solutions will be infused through the inflow until a circuit is established among the abdominal cavity, a pump and a heat exchanger. Once good flow is established, chemotherapeutic drugs will be supplemented to the perfusate and allowed to circulate in the cavity for 60 minutes. The perfusate temperature will be titrated to achieve an outflow temperature of 40-42 ^{o}C. At the end of the HIPEC procedure, the perfusion circuit is subsequently drained, the skin reopened and the abdomen inspected and lavaged with normal saline. The abdomen is closed in standard fashion and the procedure concluded.

Recurrent retroperitoneal sarcoma is confirmed with final pathology in all patients. The HIPEC drug to be used is doxorubicin only. During the HIPEC procedure, hemodynamic and cardiopulmonary parameters are continuously and carefully monitored. At the end of radical resection and HIPEC, all patients will be transferred to the intensive care unit or intermediate care area for subsequent monitoring and afterwards transferred to the surgical ward for recovery. During the surgery, we may be taking photographs of the tumour. Care will be taken to ensure that the patient will not be identified in these pictures. These pictures may be used for research publications and presentations.

4.4 Follow-up Visits

Study visits will coincide with routine clinic follow-up visits before and after the surgical procedure. After surgery, patients will be followed-up for every 2 weeks (± 10 days) for the first month and every three months (± 1 month) up to a year post-surgery for chemotherapy toxicity. Clinic visits will monitor the patient for post treatment complications as well as capture and address any adverse events, if any. At the discretion of the examining doctors, the assessments performed during the study visits include, but are not limited to, routine blood count, renal panel, and liver function tests. Any records of adjuvant treatments or treatments upon recurrence of disease will also be collected.

4.5 Survival Follow-up

After a year from resection and HIPEC, the patient will come for routine clinic review or will be contacted via telephone every year (± 1 month) for 3 years.

4.6 Discontinuation/Withdrawal

4.6.1 Discontinuation Criteria

Subjects can be discontinued or withdrawn from the study by the doctor or the Principal Investigator of this study may stop their participation in the study at any time for one or more of the following reasons:

- The Principal Investigator decides that continuing their participation could be harmful.
- Pregnancy.
- Patient requires treatment not allowed in the study.
- The study is cancelled.
- Other administrative reasons.
- Unanticipated circumstances.

4.6.2 Discontinuation Visit and Procedures

Subjects may withdraw voluntarily from participation in the study at any time. Subjects may also withdraw voluntarily from receiving the study intervention for any reason. Upon withdrawal, all data pertaining to this subject will be excluded from the study and will not be included in subsequent analyses.

If voluntary withdrawal occurs, the subject will be asked to continue scheduled evaluations according to the predetermined treatment plan with their doctors. Patients will be consistently followed up at our clinics and monitored for unforeseen toxicities that arise from our treatment.

5 CHEMOTHERAPY DRUG

5.1 Rationale for Selection of Dose

The HIPEC drug to be used in this clinical trial is doxorubicin only. Doxorubicin is an anti-tumour antibiotic that exerts cytotoxicity via topoisomerase II inhibition, DNA intercalation and formation of reactive oxygen free radicals [21]. Systemic doxorubicin has demonstrated efficacy in a wide range of sarcoma histologic subtypes and is a widely accepted standard first line agent in advanced disease [22]. Doxorubicin has several attributes that render it suitable for intraperitoneal use, including stability and synergism with hyperthermia, high AUC ratio of intraperitoneal to plasma compartment, and single pass hepatic metabolism that decreases risk of systemic toxicity [23]. The chief toxicity of concern is that of peritoneal inflammation and sclerosis and intestinal obstruction. Dose escalation studies performed by Sugarbaker and colleagues, however, revealed a dose of 15mg/m2 to be safe with regards to subsequent gastrointestinal function [24].

5.2 Study Drug Administration

Doxorubicin will be administered as an injectable solution into the abdomen.
At the end of the surgical resection, four drains will be placed in the abdominal cavity as outflow drains while one drain will be the inflow drain. Doxorubicin will be diluted into 2 – 2.5L of peritoneal dialysate fluid and circulated into the abdomen using a Belmont hyperthermia pump used by the surgical oncology team for routine intra-peritoneal chemotherapy for other pathologies. Doxorubicin will then be subsequently drained after 60 minutes. The duration of doxorubicin to be used in this study is 60 minutes. HIPEC is usually performed for 60 – 90 minutes immediately after resection is completed [23]. In our institution, HIPEC is administered for 60 minutes in all cases [25].

5.3 Storage and Drug Accountability

The study pharmacist will ensure that all investigational drugs are stored in a secured area under recommended storage conditions and in accordance with applicable regulatory requirements. Drug accountability forms will be used.

6 SAFETY MEASUREMENTS

6.1 Definitions

An adverse event (AE) is any untoward medical occurrence in a patient or clinical investigation subject administered a pharmaceutical product and which does not necessarily have a causal relationship with this treatment. An adverse event (AE) can therefore be any unfavourable and unintended sign (including an abnormal laboratory finding), symptom, or disease temporally associated with the use of a medicinal (investigational) product, whether or not related to the medicinal (investigational) product.

An elective surgery/procedure scheduled to occur during the study will not be considered an adverse event if the surgery/procedure is being performed for a pre-existing condition and the surgery/procedure has been pre-planned prior to study entry. However, if the pre-existing condition deteriorates unexpectedly during the study (e.g., surgery performed earlier than planned), then the deterioration of the condition for which the elective surgery/procedure is being done will be considered an adverse event.

A serious adverse event (SAE) is any untoward medical occurrence that at any dose:

- results in death
- is life-threatening
- requires inpatient hospitalisation or prolongation of existing hospitalisation
- results in persistent or significant disability/incapacity
- is a congenital anomaly/birth defect

6.2 Collecting, Recording and Reporting of Adverse Events and Serious Adverse Events to CIRB

All adverse events will be followed up as per institutional practice until resolution or stabilization of the event whichever comes first. All patients will be followed-up for serious adverse events for 1 year following HIPEC procedure.

Reporting of adverse events involves the Principal Investigator submitting to the approving CIRB the completed SAE Reporting Form to CIRB
within the stipulated timeframe. The Principal Investigator is responsible for informing the institution representative, the chairman medical
board (when required by the institution (for local SAE resulting in death), sponsor or regulatory bodies as required and appropriate.

regulations, will be reported to both the Ethics Committees and Regulatory Agencies as required by local regulations.

6.3 Collecting, Recording and Reporting of Serious Adverse Events (SAEs) to the Health Science Authority (HSA)

All SAEs that are unexpected and related to the study drug will be reported to HSA. Please refer to the HSA website for more information on Safety Reporting Requirements for Clinical Trials.

The investigator is responsible for informing HSA no later than 15 calendar days after first knowledge that the case qualifies for expedited reporting. Follow-up information will be actively sought and submitted as it becomes available. For fatal or life-threatening cases, HSA will be notified as soon as possible but no later than 7 calendar days after first knowledge that a case qualifies, followed by a complete report within 8 additional calendar days.

6.4 Safety Monitoring Plan

Data and safety monitoring will be performed by the PI and team of Co-Is. After every operation, the clinical data such as adverse events will be monitored monthly by the PI until end of the trial. Accuracy of the data collected and data entry will also be checked every month. This is feasible due to the relatively small number of participants to be recruited.

After the first five patients have been enrolled, the PI would perform an interim safety evaluation to assess the safety of this trial before proceeding with further enrolment.

6.5 Complaint Handling

All complains will be handled by our institution according to institutional policies.

7 DATA ANALYSIS

7.1 Data Quality Assurance

Division of Clinical Trials & Epidemiological Sciences (CTE) at NCCS will be responsible for data processing, in accordance with the defined data management procedures, under the supervision of the Investigator. Database lock will occur once quality control and assurance procedures (if applicable) have been completed.

7.2 Data Entry and Storage

The data management of this study will be undertaken by the Division of Clinical Trials and Epidemiological Sciences (CTE) at NCCS. Paper Case Report Forms (pCRF) will be provided for the recording of patient data. Detailed instructions regarding pCRF will be provided in the CRF Manual. The pCRF must be signed by the investigator to confirm that they have been checked for accuracy and completeness. The study team will conduct validation checks for correctness and completeness throughout the duration of the study. The data would be stored in a database server managed by CTE.

8 SAMPLE SIZE AND STATISTICAL METHODS

8.1 Determination of Sample Size

This study is a single arm, phase 2 intervention study.

Hypothesis H0: Recurrence rate is 80%
Alternative Hypothesis H1: Recurrence rate will drop by 20 percent i.e. it is going to be an average of 60%

Alpha:0.05
Power:0.9
Null proportion: 0.8
Alternative proportion: 0.6

The two methods used in single arm phase 2 studies are fleming's approximation method and the A'hern method, and they both work by approximating the response rates of patients by a binomial distribution, and relaxing the alpha and power values. The sample size calculated using these methods keeps the exact alpha and power values within a small tolerance, that can be preset before using the many softwares available.

Using binomial methods, we arrive at a sample size of 50 patients which would allow us to calculate an effect size to show a decrease in recurrence rates by 20%.

We have further re-looked at our sample size calculation and determined that a smaller sample size of 21 patients was sufficient to obtain statistical significance for determining a prognostic effect. Using power of 0.9, alpha of 0.05, one sided test, estimated standard deviation difference of about 2.25 and the DFI difference of 1.5 months between with HIPEC and without HIPEC, the sample size required is 21.

8.2 Statistical and Analytical Plans

Statistical analysis will be performed at the completion of the study and when the data is completed. Our institution statistician will perform the analyses according to the outcomes measured.

9 DIRECT ACCESS TO SOURCE DATA/DOCUMENTS

The investigator(s)/institution(s) will permit study-related monitoring, audits and/or IRB review and regulatory inspection(s), providing direct access to source data/document.

10 QUALITY CONTROL AND QUALITY ASSURANCE

Data and safety monitoring will be performed by the PI and team of Co-Is. After every operation, the clinical data such as adverse events will be monitored monthly by the PI until end of the trial. Accuracy of the data collected and data entry will also be checked every month. This is feasible due to the relatively small number of participants to be recruited.

11 ETHICAL CONSIDERATIONS

This study will be conducted in accordance with the ethical principles that have their origin in the Declaration of Helsinki and that are consistent with the Singapore Good Clinical Practice (as defined in the ICH E6 Guideline for GCP) and the applicable regulatory requirements.
This final Clinical Trial Protocol including the final version of the Participant Information Sheet and Consent
Form, must be approved in writing by the Centralised Institutional Review Board (CIRB) and regulatory approval from Health Sciences
Authority (HSA), prior to enrolment of any patient into the study.

The principle investigator is responsible for informing the CIRB and HSA of any amendments to the protocol or other study-related documents, as per local requirement.

11.1 Informed Consent

All informed consent forms must contain the minimum elements as mandated by the ICH guidelines and local laws and regulation requirements. This document, or any modification included, will have to be approved by IRB before use.

Before recruitment and enrolment, each prospective candidate will be given a full explanation of the study, allowed to read the approved informed consent form, and be provided ample time and opportunity to ask any questions that may arise. Once all questions have been answered and the Investigator is assured that the individual understands the implications of participating in the study, the patient will be asked to give consent to participate in the study by signing and dating the informed consent form. The Investigator will provide a copy of the signed informed consent form to each patient.

If an amendment to the protocol changes the patient participation schedule in scope or activity, or increases the potential risk to the patient, the informed consent form must be revised and submitted to the IRB and HSA for review and approval or favorable opinion. The revised informed consent form must be used to obtain consent from a patient currently enrolled in the study if he or she is affected by the amendment. The revised informed consent form must be used to obtain consent from any new patients who are enrolled into the study after the date of the approval or favorable opinion of the amendment by the IRB

11.2 Confidentiality of Data and Patient Records

Division of Clinical Trials and Epidemiological Sciences (CTE) at NCCS will be responsible for data processing in accordance with the defined data management procedures, under the supervision of the Investigator. Database lock will occur once quality control and quality assurance procedures (if applicable) have been completed.

12 PUBLICATIONS

Study findings will be published according to our department and institutional policy as determined by the National Cancer Centre Singapore.

13 RETENTION OF TRIAL DOCUMENTS

Records for all participants, including CRFs, all source documentation (containing evidence to study eligibility, history and physical findings, laboratory data, results of consultations, etc.) as well as IRB records and other regulatory documentation will be retained by the PI in a secure storage facility. The records should be accessible for inspection and copying by authorized authorities. Division of Clinical Trials and Epidemiological Sciences (CTE) at NCCS will be responsible for data processing, in accordance with the defined data management procedures, under the supervision of the Investigator. Database lock will occur once quality control and quality assurance procedures (if applicable) have been completed.

14 FUNDING and INSURANCE

This trial is not initiated by industry. The costs of the standard treatment would be the cost of the radical surgery. The costs of the investigational procedure, i.e. 2D-Echocardiography,

chemotherapy (doxorubicin), HIPEC consumables, and formal urine pregnancy tests (if applicable), would be covered by the grant of the investigator (NCCRF-YR2017-JUL-PG4).

References

1. MacNeill AJ, Miceli R, Strauss DC, Bonvalot S, Hohenberger P, Van Coevorden F, Rutkowski P, Callegaro D, Hayes AJ, Honore C, Fairweather M, Cannell A, Jakob J, Haas RL, Szacht M, Fiore M, et al. Post-relapse outcomes after primary extended resection of retroperitoneal sarcoma: A report from the Trans-Atlantic RPS Working Group. Cancer. 2017; 123(11):1971-1978.
2. Hamilton TD, Cannell AJ, Kim M, Catton CN, Blackstein ME, Dickson BC, Gladdy RA and Swallow CJ. Results of Resection for Recurrent or Residual Retroperitoneal Sarcoma After Failed Primary Treatment. Ann Surg Oncol. 2017; 24(1):211-218.
3. Trans-Atlantic RPSWG. Management of Recurrent Retroperitoneal Sarcoma (RPS) in the Adult: A Consensus Approach from the Trans-Atlantic RPS Working Group. Ann Surg Oncol. 2016; 23(11):3531-3540.
4. Singer S, Corson JM, Demetri GD, Healey EA, Marcus K and Eberlein TJ. Prognostic factors predictive of survival for truncal and retroperitoneal soft-tissue sarcoma. Ann Surg. 1995; 221(2):185-195.
5. Grobmyer SR, Wilson JP, Apel B, Knapik J, Bell WC, Kim T, Bland KI, Copeland EM, Hochwald SN and Heslin MJ. Recurrent retroperitoneal sarcoma: impact of biology and therapy on outcomes. J Am Coll Surg. 2010; 210(5):602-608, 608-610.
6. Bonvalot S, Miceli R, Berselli M, Causeret S, Colombo C, Mariani L, Bouzaiene H, Le Pechoux C, Casali PG, Le Cesne A, Fiore M and Gronchi A. Aggressive surgery in retroperitoneal soft tissue sarcoma carried out at high-volume centers is safe and is associated with improved local control. Ann Surg Oncol. 2010; 17(6):1507-1514.
7. Bartlett E and Yoon SS. Current treatment for the local control of retroperitoneal sarcomas. J Am Coll Surg. 2011; 213(3):436-446.
8. Konofaos P, Spartalis E, Moris D, Athanasiou A, Dimitroulis D, Markakis C, Kostakis ID, Nikiteas N and Kouraklis G. Challenges in the Surgical Treatment of Retroperitoneal Sarcomas. Indian J Surg. 2016; 78(1):1-5.
9. Lewis JJ, Leung D, Woodruff JM and Brennan MF. Retroperitoneal soft-tissue sarcoma: analysis of 500 patients treated and followed at a single institution. Ann Surg. 1998; 228(3):355-365.
10. Pasquali S and Gronchi A. Neoadjuvant chemotherapy in soft tissue sarcomas: latest evidence and clinical implications. Ther Adv Med Oncol. 2017; 9(6):415-429.
11. Issels RD, Lindner LH, Verweij J, Wust P, Reichardt P, Schem BC, Abdel-Rahman S, Daugaard S, Salat C, Wendtner CM, Vujaskovic Z, Wessalowski R, Jauch KW, Durr HR, Ploner F, Baur-Melnyk A, et al. Neo-adjuvant chemotherapy alone or with regional hyperthermia for localised high-risk soft-tissue sarcoma: a randomised phase 3 multicentre study. Lancet Oncol. 2010; 11(6):561-570.
12. Angele MK, Albertsmeier M, Prix NJ, Hohenberger P, Abdel-Rahman S, Dieterle N, Schmidt M, Mansmann U, Bruns CJ, Issels RD, Jauch KW and Lindner LH. Effectiveness of regional hyperthermia with chemotherapy for high-risk retroperitoneal and abdominal soft-tissue sarcoma after complete surgical resection: a subgroup analysis of a randomized phase-III multicenter study. Ann Surg. 2014; 260(5):749-754; discussion 754-746.
13. Rossi CR, Deraco M, De Simone M, Mocellin S, Pilati P, Foletto M, Cavaliere F, Kusamura S, Gronchi A and Lise M. Hyperthermic intraperitoneal intraoperative chemotherapy after cytoreductive surgery for the treatment of abdominal sarcomatosis: clinical outcome and prognostic factors in 60 consecutive patients. Cancer. 2004; 100(9):1943-1950.
14. Baratti D, Pennacchioli E, Kusamura S, Fiore M, Balestra MR, Colombo C, Mingrone E, Gronchi A and Deraco M. Peritoneal sarcomatosis: is there a subset of patients who may benefit from cytoreductive surgery and hyperthermic intraperitoneal chemotherapy? Ann Surg Oncol. 2010; 17(12):3220-3228.
15. Salti GI, Ailabouni L and Undevia S. Cytoreductive surgery and hyperthermic intraperitoneal chemotherapy for the treatment of peritoneal sarcomatosis. Ann Surg Oncol. 2012; 19(5):1410-1415.
16. Baumgartner JM, Ahrendt SA, Pingpank JF, Holtzman MP, Ramalingam L, Jones HL, Zureikat AH, Zeh HJ, 3rd, Bartlett DL and Choudry HA. Aggressive locoregional management of recurrent peritoneal sarcomatosis. J Surg Oncol. 2013; 107(4):329-334.

17. Abu-Zaid A, Azzam A, Abuzaid M, Elhassan T, Albadawi N, Alkhatib L, AlOmar O, Alsuhaibani A, Amin T and Al-Badawi IA. Cytoreductive Surgery plus Hyperthermic Intraperitoneal Chemotherapy for Management of Peritoneal Sarcomatosis: A Preliminary Single-Center Experience from Saudi Arabia. Gastroenterol Res Pract. 2016; 2016:6567473.
18. Sardi A, Sipok A, Baratti D, Deraco M, Sugarbaker P, Salti G, Yonemura Y, Sammartino P, Glehen O, Bakrin N, Diaz-Montes TP and Gushchin V. Multi-institutional study of peritoneal sarcomatosis from uterine sarcoma treated with cytoreductive surgery and hyperthermic intraperitoneal chemotherapy. Eur J Surg Oncol. 2017; 43(11):2170-2177.
19. Sommariva A, Pasquali S, Del Fiore P, Montesco MC, Pilati PL, Rastrelli M, Niba J, Nitti D and Rossi CR. Cytoreductive surgery and hyperthermic intraperitoneal chemotherapy in patients with peritoneal sarcomatosis: long-term outcome from a single institution experience. Anticancer Res. 2013; 33(9):3989-3994.
20. Chen L and Teo MCC. Cytoreductive surgery and hyperthermic intraperitoneal chemotherapy in the management of recurrent leiomyosarcoma with peritoneal sarcomatosis: A case report and review of the literature. J Cases Obstet Gynecol. 2017; 4(1):6-10.
21. Thorn CF, Oshiro C, Marsh S, Hernandez-Boussard T, McLeod H, Klein TE and Altman RB. Doxorubicin pathways: pharmacodynamics and adverse effects. Pharmacogenet Genomics. 2011; 21(7):440-446.
22. Judson I, Verweij J, Gelderblom H, Hartmann JT, Schoffski P, Blay JY, Kerst JM, Sufliarsky J, Whelan J, Hohenberger P, Krarup-Hansen A, Alcindor T, Marreaud S, Litiere S, Hermans C, Fisher C, et al. Doxorubicin alone versus intensified doxorubicin plus ifosfamide for first-line treatment of advanced or metastatic soft-tissue sarcoma: a randomised controlled phase 3 trial. Lancet Oncol. 2014; 15(4):415-423.
23. Goodman MD, McPartland S, Detelich D and Saif MW. Chemotherapy for intraperitoneal use: a review of hyperthermic intraperitoneal chemotherapy and early post-operative intraperitoneal chemotherapy. J Gastrointest Oncol. 2016; 7(1):45-57.
24. Sugarbaker PH, Mora JT, Carmignani P, Stuart OA and Yoo D. Update on chemotherapeutic agents utilized for perioperative intraperitoneal chemotherapy. Oncologist. 2005; 10(2):112-122.
25. Tan G, Chia C, Kumar M, Choo SP, Chia J, Tham CK, Chua C, Soo KC and Teo M. 201 consecutive cytoreductive surgery (CRS) and hyperthermic intraperitoneal chemotherapy (HIPEC) procedures in a single Asian tertiary centre. Int J Hyperthermia. 2016:1-7.
26. Teo MC, Ching Tan GH, Lim C, Chia CS, Tham CK and Soo KC. Colorectal peritoneal carcinomatosis treated with cytoreductive surgery and hyperthermic intraperitoneal chemotherapy: the experience of a tertiary Asian center. Asian J Surg. 2015; 38(2):65-73.
27. Huo YR, Richards A, Liauw W and Morris DL. Hyperthermic intraperitoneal chemotherapy (HIPEC) and cytoreductive surgery (CRS) in ovarian cancer: A systematic review and meta-analysis. Eur J Surg Oncol. 2015; 41(12):1578-1589.
28. Newton AD, Bartlett EK and Karakousis GC. Cytoreductive surgery and hyperthermic intraperitoneal chemotherapy: a review of factors contributing to morbidity and mortality. J Gastrointest Oncol. 2016; 7(1):99-111.
